# Inter-Observer Agreement and Laboratory Correlation of the 4T Scoring Model for Heparin-Induced Thrombocytopenia: A Single-Center Retrospective Study and Literature Review

**DOI:** 10.3390/jcm14248692

**Published:** 2025-12-08

**Authors:** Roaa M. Aljumaa, Alhomam Dabaliz, Homaira Sabur, Ali Mushtaq, Mohammad M. Aljumaa, Hani Tamim, Tarek Owaidah, Muhammad Raihan Sajid

**Affiliations:** 1 College of Medicine, Alfaisal University, Riyadh 11533, Saudi Arabiaalmdabaliz@alfaisal.edu (A.D.);; 2 Department of Internal Medicine, Cleveland Clinic Foundation, Cleveland, OH 44195, USA; 3 Pathology and Laboratory Medicine Division, King Faisal Specialist Hospital & Research Center, Riyadh 11211, Saudi Arabia

**Keywords:** heparin induced thrombocytopenia, 4T score, scoring model, inter-observer variability, diagnostic accuracy

## Abstract

**Background**: Heparin-induced thrombocytopenia (HIT) is a rapid, life-threatening adverse drug reaction, with the widely used 4T score being the critical clinical tool guiding the need for serological confirmation. This study aimed to validate the diagnostic utility of the 4T score and evaluate its application in a tertiary hospital in Saudi Arabia. **Methods**: In this retrospective cohort study, we analyzed 449 patients with suspected HIT, comparing their initially recorded clinical 4T scores with those recalculated by trained specialists, using the anti-PF4/heparin IgG ELISA as the reference standard for HIT confirmation. **Results**: Of the 292 patients who underwent laboratory testing, the HIT positivity rate was 6.5% (*n* = 19). The primary finding was a markedly low agreement (15.4%) between the system-recorded and the standardized physician-calculated 4T scores, indicating significant inter-observer variability. Despite this, higher calculated 4T scores remained significantly associated with positive HIT test results, and the anti-PF4/heparin IgG ELISA demonstrated 100% sensitivity and 100% negative predictive value (NPV). **Conclusions**: While the 4T score is indispensable for guiding the diagnostic pathway, the observed profound inter-observer variability highlights an urgent need for standardized scoring training and protocol refinement to enhance diagnostic accuracy and reduce inappropriate therapeutic interventions.

## 1. Introduction

Heparin-induced thrombocytopenia (HIT) is a rare but serious immune-mediated adverse reaction to heparin, characterized by the formation of antibodies against platelet factor 4 (PF4) and heparin complexes [1]. This condition can lead to a significant reduction in platelet count and an increased risk of life-threatening thrombosis [2]. HIT typically occurs in 1–3% of patients receiving unfractionated heparin (UFH) and less frequently with low-molecular-weight heparin (LMWH), but its clinical presentation can be difficult to distinguish from other causes of thrombocytopenia, complicating diagnosis [3,4,5].

The challenge in diagnosing HIT stems from its nonspecific clinical manifestations, such as thrombocytopenia and thrombosis following heparin exposure, which can be seen in various hospitalized patients [6,7]. Additionally, laboratory testing, while essential, also presents limitations. Enzyme-linked immunosorbent assay (ELISA)-based assays, though highly sensitive, often lack specificity, leading to false positives [8,9]. More definitive functional assays like the serotonin release assay (SRA) offer better specificity but are available only at specialized centers, limiting their real-time utility in clinical practice [10].

To address these challenges and improve the diagnostic accuracy, the 4T scoring model has been developed as a pretest probability tool [11]. This scoring system assists physicians in assessing the likelihood of HIT at the time of initial evaluation and aids in decision-making regarding laboratory testing and treatment strategies. The scoring system categorizes patients based on the degree of thrombocytopenia, the timing of heparin administration, the presence of thrombosis and other clinical sequalae, and the likelihood of other causes of thrombocytopenia into 3 groups from low to high probability (Table 1). The 4T score is highly effective at ruling out HIT: its strong negative predictive value, especially in the low probability group, allows clinicians to confidently exclude the diagnosis without further testing [2]. However, because the model has a relatively low positive predictive value, serological confirmation is always required for patients in moderate and high probability groups.

In cases where the clinical likelihood score is low, unnecessary serologic testing should be avoided to prevent the potential harm of overdiagnosis. Over-reliance on sensitive assays without considering clinical context can lead to overdiagnosis, unnecessary treatment, and potential complications. Misinterpretation of test results can prompt the use of expensive, unfamiliar and mostly not available anticoagulants, increasing risks such as bleeding and delayed treatments [12]. Moreover, incorrect HIT diagnoses often remain in medical records, often as a heparin allergy, potentially complicating future patient management [13].

In this study, we aim to retrospectively assess whether the 4T scores initially calculated by physicians differ when recalculated in retrospect. By comparing the original 4T scores with those reassessed by trained physicians, we will evaluate interobserver variability and the correlation of both scores with laboratory-confirmed HIT diagnoses. These results aim to provide insights into the clinical effectiveness of the 4T score, offering guidance on how to improve diagnostic precision and optimize patient care by reducing variability in clinical practice. Timely and accurate diagnosis of HIT is essential for making appropriate treatment decisions, minimizing unnecessary interventions, and ensuring optimal outcomes.

## 2. Methods

### 2.1. Patients and Study Design

**Figure 1:**
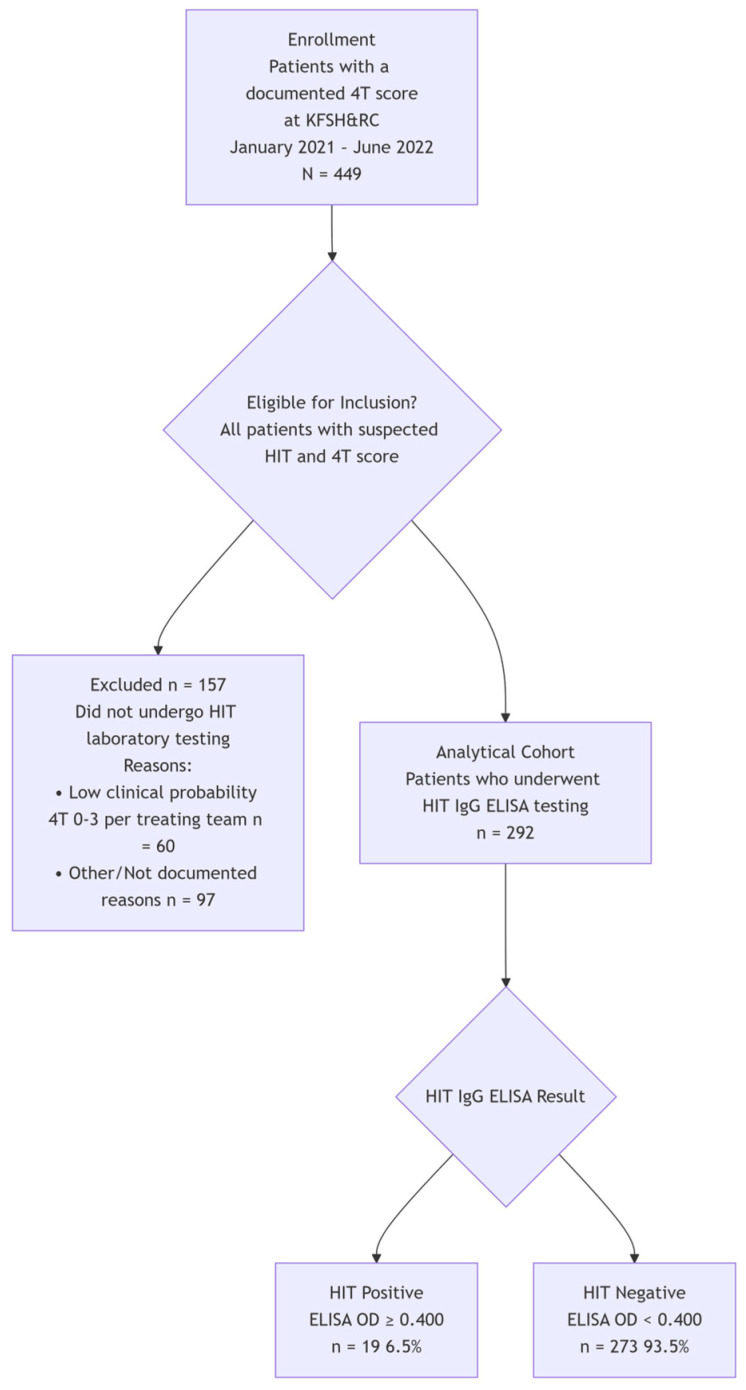
Flow diagram demonstrating the methods.

We performed a retrospective cohort chart review by searching the King Faisal Specialist Hospital and Research Center (KFSH&RC) database to evaluate the accuracy of 4T testing in accurately predicting HIT. A total of 449 patients with suspected HIT had 4T scores calculated and documented in the system between January 2021 and June 2022 were looked at. Among these patients, 292 had HIT testing performed and were included in the study. All patients outside KFSH&RC were excluded from the study. Data were collected from electronic health records, encompassing demographics, HIT Optical Density (OD), final diagnosis, platelet count, and heparin type. To clearly understand the objectives of our study, we calculated the 4T score using specific criteria found below in Table 1. The methods are shown in a flow diagram in Figure 1.

### 2.2. Measurements

The 4T score was calculated to assess the likelihood of HIT in patients with thrombocytopenia who have been exposed to heparin. The score is based on four clinical features: development of thrombocytopenia, timing of the drop in the platelet count, development of thrombosis after heparin administration, and presence of any other clinical explanation for thrombocytopenia. To calculate the 4T score, each of the four clinical features is assigned a score of 0, 1, or 2, depending on the severity of the feature. The scores are then added together to obtain a total score, which ranges between 0 and 8 with scores of 0–3, 4–5, 6–8 classified as low, intermediate, and high probability of HIT, respectively. The higher the score, the greater the likelihood of HIT [2]. HIT antibodies were confirmed using a commercial IgG-specific enzyme-linked immunosorbent assay (ELISA) (Hyphen BioMed ZCup-GTI Diagnostics, according to the manufacturer’s instructions). The test was considered positive at an optical density (OD) value of ≥0.400. Positivity was further stratified as follows: low positive (OD 0.400–1.000), moderate positive (OD 1.000–2.000), and strong positive (OD > 2.000).

To assess interobserver agreement, two physicians (M.R.S. and T.O.), both specialists in hematology, were trained on the 4T scoring model using the original criteria [11]. The training consisted of a standardized one-hour session reviewing the scoring components with example cases to ensure consistent interpretation. These physicians then independently recalculated the 4T scores for all 449 patients based on a structured data extraction form from the electronic health records. Crucially, they were blinded to both the original system-recorded 4T scores and the results of the HIT IgG ELISA during this process. The inter-rater reliability between these two trained physicians was substantial, with a Cohen’s Kappa of κ = 0.78 for the three probability categories (low, intermediate, high). Any discrepancies in their initial independent scores were adjudicated through a consensus discussion to produce a single ‘standardized physician-calculated’ score for each patient, which was used for all subsequent analyses.

### 2.3. Statistical Analysis

Statistical analyses were conducted using SPSS version 29.0. Inter-observer agreement between the system-recorded and standardized physician-calculated 4T score categories was assessed using Cohen’s Kappa (κ). The diagnostic performance (sensitivity, specificity, positive predictive value [PPV], and negative predictive value [NPV]) of both scoring methods was calculated against the IgG ELISA result, with 95% confidence intervals (CI) derived from exact binomial methods. Other analyses included cross tabulations, chi-square tests, and descriptive statistics. The chi-square test assessed the association between “Heparin Type” and “Final Dx.” Descriptive statistics, as presented in Table 2, described the central tendencies and variability of key variables, including “HIT OD,” “PLT Count at time of Dx of HIT test,” and “Age”.

## 3. Results

### 3.1. Demographics and Patient Characteristics

The study cohort consisted of 151 (51.7%) male and 141 (48.3%) female patients, with a mean age of 56.4 years (ranging from 1 to 100 years). The mean platelet counts at the time the HIT test was 72,900/µL (ranging from 3 to 313). Of the total cohort, 280 patients (95.9%) had received unfractionated heparin (UFH), while only 12 patients (4.1%) had received low-molecular-weight heparin (LMWH).

### 3.2. Comparison of Calculated 4T Scores and Agreement

A notable discrepancy was observed between the calculated 4T scores by trained physicians and those recorded in the system by practicing physicians. Table 1 provides insight into the distribution of the 4T scores, their interobserver agreement, and their correlation with system results. The highest interobserver agreement was seen for 4T scores of 4 and 5, constituting nearly 50% of all results. Only 15.4% of cases exhibited agreement between the two sets of calculations, suggesting variation in the interpretation and application of the 4T scoring model among healthcare professionals. The inter-observer agreement between the system-recorded and standardized physician-calculated 4T score categories was poor, with a Cohen’s kappa of κ = 0.12 (95% CI: 0.08–0.16).

The diagnostic performance of the two scoring methods, using a score of ≥4 as a positive test threshold, is summarized in Table 3. The standardized physician-calculated score demonstrated a higher specificity (75.1% vs. 71.8%) and PPV (17.1% vs. 14.8%) compared to the system-recorded score, while the system-recorded score showed a higher sensitivity (78.9% vs. 63.2%).

### 3.3. Positive HIT Diagnoses and 4T Scores

Cross-tabulation of 4T scores (Table 4) calculated by different physicians and the final diagnosis indicated that many patients with 4T scores of 4 were ultimately diagnosed as “Negative.” This finding suggests that a 4T score of 4 may align with a lower risk of HIT. The most common diagnosis for 4T scores of 0 and 1 was “HIT is not indicated,” highlighting alignment between the 4T scoring model and clinical diagnosis in these cases. Out of 292 tested samples, 19 (6.5%) received positive HIT diagnoses, with varying levels of positivity. Among these positive cases, 11 cases (3.8%) were classified as low positive for HIT, 6 cases (2%) were moderate positive, and 2 cases (1.3%) were strongly positive.

The distribution of calculated 4T scores among positive HIT diagnoses suggests that moderate and high 4T scores were associated with positive HIT cases. Specifically, out of the 19 positive HIT cases, 12 cases (63.2%) had moderate and high calculated 4T scores by trained physicians, while 17 cases (89.5%) had moderate and high calculated 4T scores recorded in the system calculation.

### 3.4. Comparison of Calculated 4T Scores and Potential Missed Cases

Examining the calculated 4T scores for the 449 patients with suspected HIT, we found that 142 (31.6%) had a system-recorded 4T score of less than 4, while 136 (30.3%) had a standardized physician-calculated score of less than 4. Of the initial 449 patients, 157 did not undergo HIT laboratory testing. Among these 157, the most common reason for not testing was a low clinical probability (a system-recorded 4T score of 0–3) as determined by the treating team, which accounted for 60 cases (38.2% of the untested group). Furthermore, based on the standardized recalculation, 7 of the 19 confirmed HIT-positive cases (36.8%) had a system-recorded score in the low-probability range (0–3), indicating they could have been missed if reliance was placed solely on the initial clinical score.

#### Positive HIT Diagnoses and 4T Scores

Out of 292 tested samples, 19 (6.5%) were positive for HIT. The distribution of these positive cases according to both scoring methods is presented in Table 4. According to the system-recorded scores, 15 of the 19 positive cases (78.9%) were in the moderate/high probability categories (4–8). In contrast, the standardized physician-calculated scores placed 12 of the 19 positive cases (63.2%) in the moderate/high probability categories.

### 3.5. Heparin Type and HIT

No significant association was found between the type of heparin used (UFH or LMWH) and the final diagnosis of HIT.

## 4. Discussion

### 4.1. Overview and Applications

Our single-center retrospective study evaluated the real-world application and diagnostic performance of the 4T scoring model for HIT. The primary finding was a notably low inter-observer agreement (κ = 0.12) between the 4T scores initially recorded by treating physicians in the clinical system and those recalculated in a standardized, blinded manner by trained hematology specialists. This profound variability underscores a significant challenge in the consistent application of the 4T score in clinical practice.

This discrepancy in scoring had tangible clinical implications. We found that 7 of the 19 confirmed HIT-positive cases (36.8%) had a system-recorded score in the low-probability range (0–3), suggesting they could have been missed if laboratory testing was solely guided by the initial clinical assessment. This finding aligns with known limitations of the 4T score, where its high sensitivity and negative predictive value (NPV) are contingent upon correct application [3,13]. In our cohort, the standardized physician-calculated score demonstrated a higher specificity (75.1% vs. 71.8%) and PPV (17.1% vs. 14.8%) compared to the system-recorded score. However, this came at the cost of lower sensitivity (63.2% vs. 78.9%), reflecting the inherent trade-off in clinical prediction rules and highlighting how interpretation variability can directly impact test performance metrics.

### 4.2. Effectiveness, Limitations, and Clinical Management for HIT

The 4T score was originally designed as a real-time pretest probability tool that relies heavily on the availability and accurate interpretation of sequential platelet counts and clinical context [11,12,13,14]. The poor agreement we observed likely stems from the subjective nature of certain criteria, particularly the “Timing of platelet count fall” and “Other causes for thrombocytopenia,” especially in complex, hospitalized patients where multiple causes for thrombocytopenia often coexist. Our results are consistent with previous literature acknowledging inter-observer variability as a key limitation of the 4T score [7]. The substantial inter-rater reliability (κ = 0.78) achieved by our two trained physicians after a standardized training session suggests that targeted education and clear protocols could mitigate this variability and improve the uniformity of its application.

## 5. Study Limitations and Challenges

Our study has several important limitations. Its retrospective, single-center design may limit the generalizability of our findings. The exclusion of 157 patients who were not tested for HIT, primarily based on low clinical suspicion, introduces a potential selection bias. Furthermore, the use of a retrospectively applied, physician-calculated score as our comparator, while methodologically rigorous with blinding and adjudication, inherently lacks the real-time clinical context available to the treating physician. Therefore, our ‘standardized’ score may not perfectly represent the ideal ‘gold standard’ for a bedside assessment tool. Finally, our laboratory standard was an IgG-specific ELISA. While highly sensitive, it is not the functional gold standard (Serotonin Release Assay). The lack of SRA confirmation means we cannot definitively distinguish between true HIT and clinically insignificant anti-PF4/heparin antibodies in some ELISA-positive cases.

## 6. Conclusions

In conclusion, we found significant variability between 4T scores applied in clinical practice and those recalculated in a standardized, blinded fashion. This discrepancy highlights a potential for misclassification in real-world settings. While our findings suggest that standardization and training could reduce this variability, they also underscore the inherent challenges of retrospective validation of a clinical tool. Future prospective studies are needed to confirm whether standardized training directly improves diagnostic accuracy and patient outcomes.

## Figures and Tables

**Table 1:** 4T score for estimating the pretest probability of HIT.

	2 Points	1 Point	0 Point
**Thrombocytopenia**	PLT decrease >50% AND nadir ≥ 20,000/µL AND no surgery within preceding 3 days	PLT decrease > 50% BUT surgery within preceding 3 days OR any combination of PLT fall and nadir that does not fit criteria for 2 or 0 points (eg, 30 to 50% fall or nadir 10,000 to 19,000/µL)	PLT decrease < 30% OR nadir < 10,000/µL
**Timing of onset after heparin exposure**	5 to 10 days OR 1 day if exposure within past 5 to 30 days	Probable 5 to 10 days (eg, missing PLT counts) OR > 10 days OR < 1 day if exposure within past 31 to 100 days	<4 days without exposure within past 100 days
**Thrombosis or other clinical sequelae**	Confirmed new thrombosis, skin necrosis, anaphylactoid reaction, or adrenal hemorrhage	Suspected, progressive, or recurrent thrombosis, skin erythema	None
**Other causes for thrombocytopenia**	None	Possible (eg, sepsis)	Probable (eg, DIC, medication, within 72 h of surgery)
**Interpretation**			
0 to 3 points—Low probability (<1%) 4 to 5 points—Intermediate probability (approximately 10%) 6 to 8 points—High probability (approximately 50%)			

**Table 2:** Patient characteristics that underwent HIT testing (*n* = 292).

Gender (%)		
Males	151 (51.7)	
Females	141 (48.3)	
Heparin type (%)		
Unfractionated heparin	280 (95.9)	
Low molecular weight heparin	12 (4.1)	
Clinical Characteristics		
HIT results	Positive	Negative
Age, mean (SD)	53.2 (17.3)	56.6 (19.6)
HIT OD, mean (SD)	1.20 (0.72)	0.13 (0.06)
Platelet count at time of diagnosis, mean (SD)	61.6 (41.2)	73.6 (52.4)
Normal platelet count (%)	1 (0.3)	19 (6.5)
Thrombocytopenia (%)	18 (6.2)	254 (87)
4T by Trained physicians		
0–3 (%)	7 (37)	68 (24.9)
4–5 (%)	7 (37)	139 (50.9)
6–8 (%)	5 (26.3)	66 (24.2)
4T by System generated		
0–3 (%)	4 (21)	47 (17.3)
4–5 (%)	3 (16)	149 (54.6)
6–8 (%)	12 (63)	77 (28.3)

**Table 3:** Diagnostic Performance of the 4T Scoring Methods (Using a Score of ≥4 as a Positive Test).

Diagnostic Measure	System-Recorded Score	Standardized Physician-Calculated Score
Sensitivity	78.9% (54.4–93.9%)	63.2% (38.4–83.7%)
Specificity	71.8% (66.1–77.0%)	75.1% (69.5–80.1%)
Positive Predictive Value (PPV)	14.8% (8.5–23.3%)	17.1% (9.9–26.6%)
Negative Predictive Value (NPV)	98.2% (95.2–99.5%)	96.0% (92.5–98.1%)

**Table 4:** Distribution of HIT-Positive Cases by 4T Score Category and Scoring Method.

4T Score Category	HIT Positive (*n* = 19)	HIT Negative (*n* = 273)	Total Tested (*n* = 292)
**Trained Physicians’ Calculation**			
Low (0–3)	7 (36.8%)	68 (24.9%)	75 (25.7%)
Intermediate (4–5)	7 (36.8%)	139 (50.9%)	146 (50.0%)
High (6–8)	5 (26.3%)	66 (24.2%)	71 (24.3%)
**System-Recorded Calculation**			
Low (0–3)	4 (21.1%)	47 (17.2%)	51 (17.5%)
Intermediate (4–5)	3 (15.8%)	149 (54.6%)	152 (52.1%)
High (6–8)	12 (63.2%)	77 (28.2%)	89 (30.5%)

## Data Availability

Data is available from authors on request.
